# An Immediate Fit, Adjustable, Modular Prosthetic System for Addressing World-Wide Limb Loss Disability

**DOI:** 10.1016/j.arrct.2021.100120

**Published:** 2021-03-15

**Authors:** Jessica Kenia, Bethany Wolf, Jim Marschalek, Timothy Dillingham

**Affiliations:** a Department of Physical Medicine and Rehabilitation, University of Pennsylvania School of Medicine, Philadelphia, PA; b Friends of the Redeemer United, St. Elizabeth, Jamaica; c Advanced Design Concepts, Pewaukee, WI

**Keywords:** Amputation, Case report, Rehabilitation

## Abstract

Prosthetic services and resources globally are insufficient to meet the needs of individuals with lower limb loss worldwide, particularly in low resource countries. The lack of trained prosthetists, high cost, and inaccessibility of prosthetic services leave many patients in these countries without a prosthesis. To address this problem, an immediate fit, adjustable, modular, prosthetic system was developed. Six individuals in Jamaica with transtibial lower limb loss who were in need of a prosthesis visited a local therapy clinic. They were fit with the prosthetic system by a physical therapist certified and trained in the proper fitting of these devices. All patients were fit on the first visit and walked out with a comfortable prosthesis after some rehabilitation gait training. Five of the patients returned for follow-up and reported that they continued to use these devices for daily wear. No adverse events or socket component failures were reported despite rigorous daily use in a rugged environment. This new prosthetic care delivery model—a modular system distributed to patient locations and fit by trained allied rehabilitation professionals—holds potential for meeting the large demand for lower limb prosthetics in developing countries.

## Highlights

• The iFIT prosthesis can be fit in 1 session.
• iFIT prostheses are modular and easily transported in bulk.
• Only a few hand tools are necessary to fit and align these devices.
• They can be fit in austere settings, including rural hospitals and general clinics, a patient's home, or any mobile medical facility.
• Interested allied medical personnel can be successfully trained in how to fit and align the iFIT prosthesis. This greatly expands the pool of providers across a region or country.
• The iFIT devices are waterproof and durable enough to last years. This alleviates the need for repeated socket fabrication and adjustments, costly prospects for patients with limited resources to travel.
• iFIT sockets adjust and accommodate growth in teens.

Prosthetic services and resources worldwide are insufficient to meet the needs of individuals with lower limb loss particularly in low resource countries.^1^ Such a disabling condition has profound negative effects on the ability of these individuals to work, go to school, and thrive within the cultural boundaries of their society.^2^

In Jamaica, there are only a few prosthetists located on the island, and travel to these locations can be difficult and expensive. For individuals with a lower-limb amputation, obtaining a functional prosthetic device is very unlikely and consequently limits many to using crutches for ambulation. In its 2005 report, the World Health Organization estimated that 75% of developing countries lack orthotic and prosthetic training programs to meet the demands of their populations, resulting in poor access and low availability of prosthetic devices and services.^1^ There is a large and important unmet need for an economical prosthetic system for individuals with limb loss in Jamaica and other low- and middle-income countries.

iFIT Prosthetics, LLC developed a mass produced transtibial prosthesis^a^ to efficiently meet the needs of patients in low resource countries. These prostheses were all tested for strength and durability according to the International Organization for Standardization and are suitable as initial prostheses or permanent devices. This “prosthesis in a box” was developed for such international assistance. This prosthesis has been clinically tested in the United States and has been shown to be comparable to conventional sockets in self-reported satisfaction and comfort, as well as reduced intrasocket pressures on the residual limb.^3^^,^^4^ These prostheses are commercially available and can be shipped anywhere in the world with the components necessary for fitting patients. The only additional items needed for the patient are a silicone sleeve for pin suspension and an appropriate prosthetic foot.

Allied health care professionals, as described in this case study, can be trained in prosthetic fitting. Training materials, including instructional videos and an instructional manual, are located online for qualified health professionals to access after registering with the company. The design of this adjustable prosthesis is forgiving, allowing individuals with a variety of lengths and limb circumferences to use these sockets. The device can accommodate patients with residual limb lengths (measured based of patella to distal end) of 14-20 cm with a standard device and up to 25 cm with a tall device. This is accomplished by using spacers that were designed to be used specifically with this system to position the limb in the optimal position in the device. The standard and wide devices that were sent to Jamaica can accommodate residual limb circumferences from 24-39 cm. There are 6 commercially available sockets of differing sizes that can accommodate both larger and smaller circumferences.

**Fig 1:**
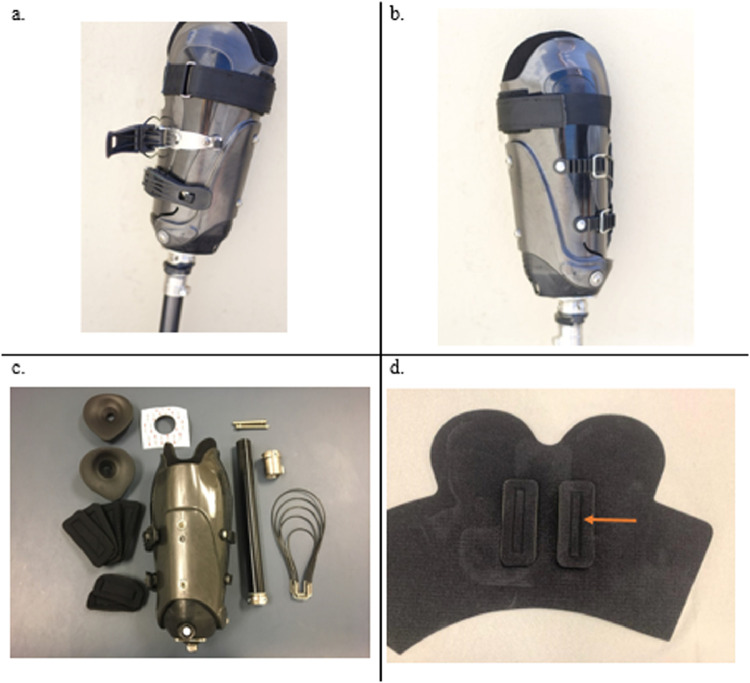
Components of the immediate fit prosthesis. (A) Lateral view of prosthesis displaying locking buckle system; (B) medial view of prosthesis displaying hooks used to make fine adjustments to the socket's circumference; and (C) the “prosthesis in a box” kit including a socket, neoprene liner, padding kit, spacers, adhesive, pylon, tube clamp, pins, and various sized cables. All that is needed in addition to the kit is a sleeve with pin attachment (pins are included) for suspension and a prosthetic foot. (D) A 5-mm perforated neoprene liner shown with additional pads (arrow) that stick to the liner with velcro and can be placed to relieve socket pressure and reduce discomfort. These pads allow for a customized socket fit.

This adjustable, immediate fit prosthetic system differs from conventional prosthetics in several important ways (fig 1). A locking buckle system and flexible socket provides adjustability and a more comfortable fit.^3^^,^^4^ A soft padded inner liner can be customized and provides relief over boney prominences. The socket is a total contact design, which helps manage and prevent limb edema.

## Case study

This study was reviewed and approved by the University of Pennsylvania Institutional Review Board (protocol no. 829742). The participants all gave verbal consent to be fit with the prosthetic device. The prosthetics in this case study were donated by iFIT Prosthetics, LLC to help individuals with limb loss who lacked a well-fitting prosthetic device owing to cost, travel, or poor fit. Patients were fit with the prosthesis by an experienced physical therapist who was employed by a local clinic and was trained to fit the device by the principal investigator (PI) (T.D.). The patients were included if they met the following criteria: transtibial amputation, well healed limb, weight under 260 pounds, and no stroke or brain injury that could affect safe walking.

Six individuals with transtibial limb loss were referred to the physical therapist and fit with the prosthesis. The patients were first screened for inclusion criteria. They had their blood pressure, weight, and heart rate recorded during each session. They were then fit and aligned with the prosthesis during a single session. Each was given gait training and instructed on how to adjust the system with the locking buckle system. All patients were followed for periods of time ranging from 7 months to 2 years. During the follow-up period, patients were given a questionnaire consisting of 14 questions, each with 5 levels of response for a 70-point total prosthetic satisfaction questionnaire based on the Prosthetic Evaluation Questionnaire.^5^ They rated the prosthetic device in terms of comfort, stability, and adjustability from 1 (poor)-5 (excellent).

To fit the prosthesis, the device was buckled onto the individual's residual limb so that it was snug. The pylon was cut to the appropriate length and attached to the prosthesis and a prosthetic foot using a 4-mm Allen wrench. The therapist then aligned the prosthesis for walking. Each prosthesis was customized to the patient by having additional pads placed in areas of high pressure. Additional modifications, such as heating the prosthesis and trimming the brim for shorter limbs, were not needed.

To ensure the prosthetic was being properly fit, the therapist kept a log of all notes from each session and took pictures of the participant's residual limb and prosthesis once fit. She also took video of the participant's gait during their initial fitting and follow-up sessions. The images and videos were securely shared with the PI to ensure that proper fitting techniques were being used. Each participant had their residual limb inspected for skin breakdown and was instructed to routinely check themselves after walking and report any issues promptly. Any adverse events were to be reported to the PI. A summary of the participants and their ratings are presented in table 1.

**Table 1:** Characteristics of 6 patients fit with the immediate fit transtibial prosthesis

Patient No.	Age, y	Length of Time Between Amputation and iFIT	Etiology	Limb Measures	Length of Time Wearing the iFIT	Rating of iFIT (Out of 70)	Previous Socket
1	40	15 y	Trauma	Length: 14 cm Circumference: 29 cm	2 y	66	None, ambulated with crutches
2	27	2 y	Trauma	Length: 18 cm Circumference: 28 cm	1 y, 4 mo	51	None, ambulated with crutches
3	12	4 y	Congenital	Length: 14 cm Circumference: 24 cm	11 mo	61	None, ambulated with crutches
4	36	7 y	Trauma	Length: 14 cm Circumference: 29 cm	Lost to follow-up	-	None, ambulated with crutches
5	63	2 y	Diabetes/ vascular	Length: 16 cm Circumference: 33 cm	7 mo	59	None, ambulated with crutches
6	43	3 mo	Diabetes/ vascular	Length: 18 cm Circumference: 38 cm	1 mo	64	Icecast prosthesis, rated at 19 points; this socket became uncomfortable when the patient's limb shrank in size

In this case series, all participants were able to fit in the standard size socket. Several other sizes have been developed for larger limbs, but these were not needed for the patients seen in this case series. The range of adjustment on each device was enough to accommodate each individual's need for adjustability as indicated during follow-up visits.

All patients achieved safe, independent ambulation without crutches during the first session after fitting. The first patient had not walked with a prosthesis for 15 years and was able to walk unassisted with the immediate fit device after gait training. After the fitting, he took an interest in helping the therapist with future fittings and learning how to counsel and instruct other patients in the use of these prostheses.

**Fig 2:**
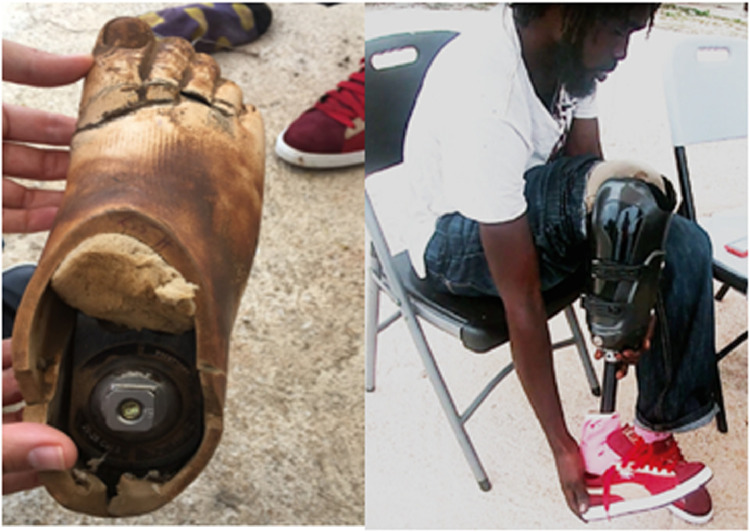
The prosthetic foot (left) showing breakdown of the outer cover after 6 months of use. The iFIT socket (right) remained in working order over 7 months after being used extensively while performing this individual's occupation (agricultural work).

**Fig 3:**
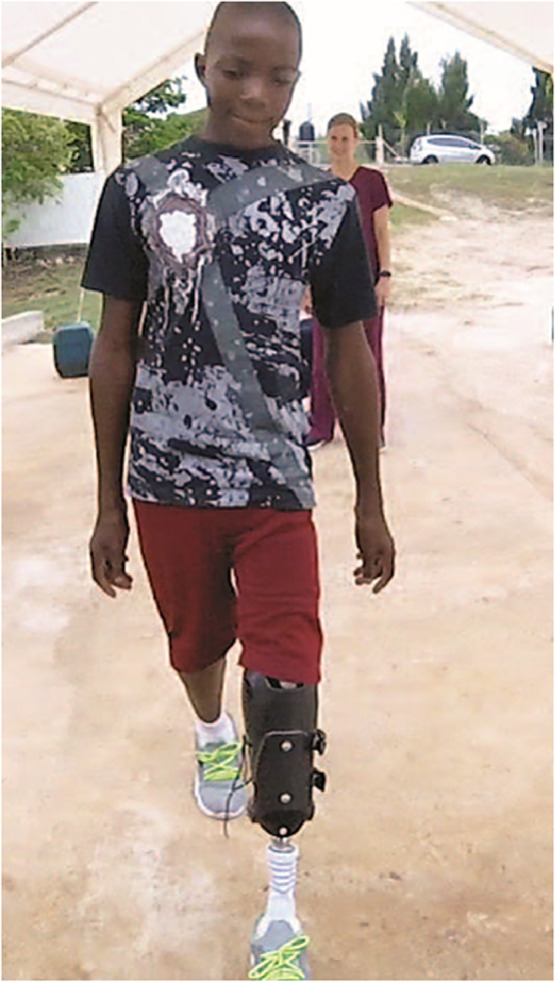
The iFIT prosthesis was successfully fit on a 12-year-old boy. It accommodated his growth.

One patient had a physically demanding occupation in agriculture. During a follow-up appointment 7 months after being fit, the prosthesis remained quite functional and without mechanical breakage. The prosthetic foot and liner had degraded significantly, however, owing to the climate and occupational wear and required replacement (fig 2). According to the therapist, this is a common problem with medical equipment subjected to the climate in Jamaica. One patient was a teenager with congenital limb deficiency who was 12 years old when fit. He has worn the prosthesis for approximately 1 year and reported that it accommodated his growth and remained comfortable and functional (fig 3). His father remarked that he was able to get around school much better and that the prosthetic has benefitted his son greatly.

There was only 1 patient who had used a conventional (hard) socket for a short period before using our device. This socket was quickly abandoned as his limb changed in size and it became uncomfortable. This patient reported that he was only able to wear his previous prosthesis for 1 to 3 hours per day. During a follow-up phone call 1 month later, he reported wearing the immediate fit, adjustable prosthesis for the entire day (≥14h) without any complications such as skin breakdown. This patient also remarked that the adjustable prosthesis felt “cooler” and had better temperature regulation. Only 1 patient in this group did not return for follow-up assessment. In summary, the immediate fit, adjustable prostheses were successfully fit and fully utilized by 6 active patients in Jamaica for extended periods of time.

## Discussion

This case series of Jamaican individuals with transtibial limb loss demonstrated that the iFIT transtibial prosthesis is a useful, feasible, and functional prosthetic device for individuals in a country with limited prosthetic services. A physical therapist was successfully trained in fitting and aligning these prostheses. Training allied health professionals, technicians, and medics to use this device could be a means of providing prosthetic devices in an efficient manner across wide geographic (rural) areas in countries with few prosthetic services.

These immediate fit, fully adjustable devices appear to be sufficiently durable as evidenced by this cohort of patients. They are waterproof and composed of strong materials that withstood the daily rigors of rural life in Jamaica. This means very little or no follow-up maintenance is needed and frequent visits, which are costly for these individuals, to prosthetic facilities (when available) is largely unnecessary. An adjustable socket can be very useful to accommodate expected changes in residual limb shape and volume, or in the case of a teenager, expected normal growth. These sockets are composed of injection molded polymer materials that offer high quality and consistency at more accessible and affordable price. In the United States, the cost is approximately one-fifth of the price of a traditional prosthesis, depending on the componentry.^6^

This prosthesis is also waterproof, which is an important characteristic for people living in wet environments. Long-term durability of a socket is an essential factor to consider as the rural areas of Jamaica and other low resource countries also feature rugged terrain, dirt roads, and long distances for people to travel for prosthetic repairs or modifications. The ability of the prosthetic user to make any adjustments themselves provides a high level of independence and freedom from the need for frequent socket modifications (typical for hard conventionally made sockets).

The 12-year-old patient described previously illustrated a major advantage of the adjustable sockets—patient-centered adjustability. Having a prosthesis had a positive psychological effect for this teenager. He still uses the same socket now after almost a year, and it has readily accommodated his growth. Teens using conventional sockets require frequent adjustments and new sockets every 6 months or sooner as they grow.^7^ For growing children with limb loss in low resource countries, an adjustable prosthesis is optimal to keep them comfortable and at their maximum functional level over time.

Five of the patients fit with the immediate fit prosthetic system did not have a prosthesis. They all ambulated with 2 axillary crutches, illustrating the profound disability of limb loss for these individuals. Disability has been shown to increase poverty, leading to increased economic and social disadvantages.^8^ The 4 men of working age in this study all reported that being able to return to work was life changing for them and their families.

## Conclusions

The iFIT prosthesis system and this novel care delivery model—training allied professionals to fit and align the devices in their outpatient clinics in the patients’ communities—could prove quite useful in meeting the current and future prosthetic demands worldwide. An adjustable transfemoral system is now available, and future investigations will examine this system for patients in developing countries. Future research should involve a larger cohort of participants comparing the iFIT system to other low-cost immediate-fit options to further assess the safety, durability, and comfort of this system. In summary, the iFIT transtibial system proved to be effective in providing a comfortable and durable prosthetic device for individuals with transtibial lower limb loss in Jamaica.

## Supplier

a. TT200 Transtibial prosthesis; iFIT Prosthetics, LLC.
